# The Biomechanical Impact of Loss of an Implant in the Treatment with Mandibular Overdentures on Four Nonsplinted Mini Dental Implants: A Finite Element Analysis

**DOI:** 10.3390/ma15238662

**Published:** 2022-12-05

**Authors:** Cristian Teodorescu, Elena Preoteasa, Cristina Teodora Preoteasa, Cătălina Murariu-Măgureanu, Ioana Monica Teodorescu

**Affiliations:** 1 Department of Prosthodontics, Faculty of Dentistry, “Carol Davila” University of Medicine and Pharmacy, 020021 Bucharest, Romania; 2 Department of Scientific Research Methods-Ergonomics, Faculty of Dentistry, “Carol Davila” University of Medicine and Pharmacy, 020021 Bucharest, Romania

**Keywords:** overdenture, mini dental implants, thermo-polymerizable acrylic resin injected under pressure, finite element method

## Abstract

The aim of this study was to evaluate the biomechanical impact, in terms of stress and displacement, at the level of a mandibular overdenture, on four mini dental implants (MDIs) after the loss of an implant. A three-dimensional virtual model was obtained by scanning the overdenture, and a biomechanical analysis was carried out, using the finite element method (FEM). The displacements of the overdenture and the equivalent von Mises stresses were evaluated using logarithmic scales. In the case of a mandibular overdenture on four MDIs inserted in the interforaminal area, the frontal loading generated the lowest values for the von Mises stresses, and the bilateral loading generated the least displacement when two implants were inserted in the canine area and two in the molar area. The highest von Mises stress was observed during frontal loading in the situation of the mandibular overdenture on four MDIs, two of which were inserted in the canine area and two in the molar area, following the loss of an implant in the canine area. The largest displacement was noted in the mandibular overdenture on four interforaminal MDIs during unilateral loading, following the loss of a distally inserted implant. The FEM analysis showed aspects that correlated with clinical observations, with predictive value. The concentration of von Mises stresses, and the occurrence of some displacements of the prosthodontic restoration, can explain the emergence of some complications in the overdenture’s biodynamics, and the increased risk of fracture. Complications can be prevented by choosing a certain number of implants and a topographical distribution correlated with biomechanical aspects, and by proposing a correct occlusal scheme with optimal functional loading.

## 1. Introduction

Increasing life expectancy and falling mortality have contributed to the estimated world population growth to eight billion people in 2022. With global life expectancy in 2019 at nearly 73 years [1], the risk of occurrence of complete edentulism may increase in an aging society. Complete edentulism, as a clinical situation, is accompanied by a series of morphological and functional changes at the oro-facial level. In the current context, in order to compensate for functional and tissue changes, and to replace missing teeth, in cases of completely edentulous patients, several prosthodontic treatment options are indicated: namely, conventional complete dentures, fixed prostheses or overdentures on implants [2].

Prosthodontic treatment has positive effects on restoring the functions of the dento-maxillary apparatus, and can influence the general health, nutritional status and psychological comfort of completely edentulous patients [2]. Conventional complete dentures, especially mandibular ones, are more frequently associated with the occurrence of complications related to lack of stability or maintenance [3]: thus, two or more implants may be chosen, which can have beneficial consequences for stability, and may increase the level of patient satisfaction [4]. Overdentures on implants, due to the benefits they offer compared to conventional complete dentures, are considered the first therapeutic option in complete mandibular edentulism [2,5,6].

The diversity of overdentures, in relation to implants, is generous, and varies from those on conventional implants to those on MDIs: they can be single-piece or they can be composed of two components; they can have a reduced diameter, between 1.8 and 3 mm, and variable lengths from 10 to 18 mm, and they can be inserted using minimally invasive techniques, in a single surgical step and with immediate loading [7]. MDIs overdenture, as a way to stabilize complete dentures, especially mandibular ones, has advantages for elderly people who are completely edentulous, forming a category of vulnerable persons [8]: in fact, MDIs are an alternative that increases the quality of life of these patients. One of the predictive parameters in this regard is the mastication capacity of mandibular overdenture wearers [9].

The anatomical limitations of the alveolar ridge, which are related to severe resorptions, may contraindicate conventional implant treatment [10]; pre-implantation procedures, such as residual ridge augmentation, guided tissue regeneration, vertical distraction osteogenesis or ridge split interventions, may be necessary. The use of MDIs does not require such interventions, and ensures the long-term stabilization of overdentures [11], as the minimum width required to insert these implants is 4–5 mm [12].

The therapeutic success of implants is quantified by a clinical and radiological assessment of implant and peri-implant tissues [13], and the loss of an implant can be a possible complication in this therapeutic option. Supplementation of the number of implants and anchoring systems can have a positive impact on the retention of the overdenture, and can relieve the stresses. Several studies have shown a link between implant loss and reduced bone density, with impaired primary stability and a decreased survival rate of dental implants [14]. Researchers have found a link between a higher risk of implant loss and poor bone density: for example, in class D4, affecting implant stability [14], heavy smokers, poor oral hygiene or associated systemic conditions, such as decompensated diabetes or osteoporosis [13,14]. Additionally, there is a correlation between loss of implants due to their lack of passivity after insertion, an immediate load at a torque that is too low, incidents when the bone has a lower density, and overloading the implants. The complications of prosthodontic treatments on implants can also be generated by the lack of an adequate correlation with aspects related to the bone density, the primary stability of the implants, the prosthetic solution or the type of antagonist [15].

The cause of implant loss can be bacterial, affecting the peri-implant tissues, or mechanical, by overloading through traumatic occlusion, cyclic bending stresses or the excessive cantilever effect [16]. The failure manifests in the loss of peri-implant bone, and the appearance of mobility and radiolucency around the implants [13]. MDIs inserted in the maxillary arch have a lower survival rate than mandibular ones, and fracture of the overdenture occurs more frequently near attachment systems, especially O-ring-type systems where the thickness of the acrylic base is lower, due to their dimensions [17]. For overdenture treatment, both the conception and the correct implementation of the treatment stages are important, from the choice and insertion of the implants to the use of certain attachment systems and materials, as well as to the actual creation of the overdenture. The choice of material from which the overdenture base is made influences its resistance to the stresses developed during mastication [18]. One of the most-used materials in this regard is polymethylmethacrylate [19], which exhibits low flexural and fatigue resistance, and can cause fractures of complete dentures [20,21].

Prosthodontic manufacturing technology is also important, and the use of modern injection under the pressure techniques of thermo-polymerizable resins can ensure that more reliable dentures are obtained, in terms of bending resistance, decreasing the risk of the denture bases fracturing [22]. In situations where the overdentures’ support becomes deficient, due to the involution of bony, mucous, or muscular structures of the dento-maxillary apparatus, areas of acrylic base overload may appear near certain implants or MDIs, with an increased risk of fracture, considering that the support differs depending on the chosen implant system [23]. Through the loss of one or more implants, biomechanical changes may occur in the behavior of the overdentures, increasing the risk of complications.

The FEM allows for the simulation and evaluation of the biomechanical effects which tend to appear in overdentures on implants in the different clinical situations in which there are complications related to implants or prosthodontic restorations [24]. In this sense, the physical properties of the materials used to create the overdentures, and the loading conditions with occlusal forces, are taken into account [25,26,27].

Loss of implants and attachment systems have negative consequences on denture balance, mastication, and patient comfort. To prevent complications, the specialists recommend a correct analysis of the occlusal forces, an adequate number of implants, their optimal topographical insertion and distribution, and continuous medical care for the patient, to ensure timely and proper interventions to remedy any deficiencies that could arise. The aim of this study was to evaluate the biomechanical impact, in terms of stress and displacement at the level of a mandibular overdenture, on four MDIs after the loss of an implant. The simulated treatment alternative was a mandibular overdenture on four nonsplinted MDIs, inserted in different topographical regions. During simulation, frontal, unilateral or bilateral occlusal loadings were applied.

## 2. Materials and Methods

An in silico study was carried out using the FEM, which allows for biomechanical analysis and the evaluation of some three-dimensional geometric structures, including the tensions manifested at their level and the consequences of the application of some compressive forces. For this purpose, the physical properties of the used polymer were considered, such as the modulus of elasticity and Poisson’s ratio, and the occlusal loading conditions were simulated during incision at the level of the frontal teeth, or during the mechanical processing of food during mastication at the level of the lateral teeth.

### 2.1. Finite Element Modeling of the Mandibular Overdenture

To obtain the three-dimensional model, a mandibular overdenture was scanned by capturing a large number of images of all surfaces, from the left, right, above, below, front and back directions. An overdenture on four MDIs was chosen to be simulated because, if their reduced diameter is taken into account, the recommendations for mandibular overdentures are for four implants, not two, as in the case of conventional implants, according to the McGill and York Consensus (2002, 2009) [7].

**Figure 1:**
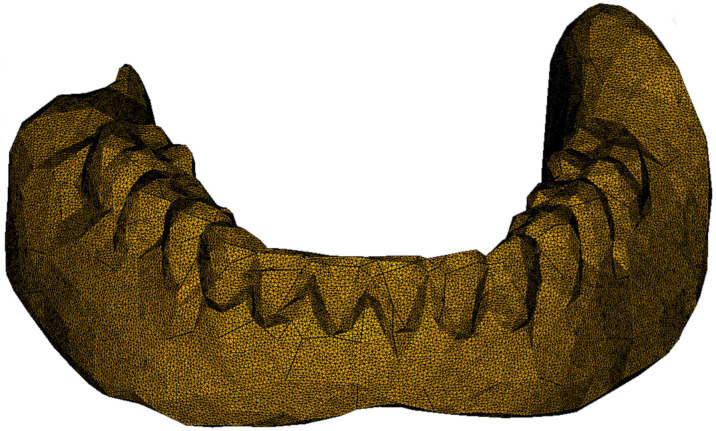
The model of a real mandibular overdenture.

The model in STL format was a network of linked triangles, which, for a large number of elements, approximates the complex shape of a mandibular overdenture. After scanning, the images were imported into the freely available open-source software, Meshmixer 3.5 (Autodesk Inc., San Rafael, CA, USA, www.meshmixer.com, accessed on 18 March 2022), in which the inherent errors resulting from scanning were corrected, to obtain surfaces that allowed for further analysis by the FEM. Using the freely available open-source software, FreeCAD (www.freecadweb.org, accessed on 18 March 2022), it was possible to import the model in STL format, check it, reduce the dimensions by reducing the number of network nodes and obtain the finite element model. The obtained results were exported to the freely available open-source software, ParaView (Kitware Inc., Clifton Park, NY, USA, www.paraview.org, accessed on 18 March 2022), which allowed their extensive presentation. The model of the mandibular overdenture is presented in Figure 1.

### 2.2. Boundary Conditions

In this study, the mandibular overdenture was made from thermoplastic polymer, namely, Vitaplex (Roko Dental Systems, Częstochowa, Poland), which was injected under pressure, using an automated injection device, Multipress Eco (Roko Dental Systems, Częstochowa, Poland). The considered mechanical properties were the modulus of elasticity E = 2684 Mpa, according to the manufacturer, and Poisson’s ratio ν = 0.28, which was characteristic of acrylic resins [28].

To obtain control of the loads, the geometry of the mandibular overdenture was slightly modified by the addition of some cylinders in the direction of application of the pressure, corresponding to the position of the implants, and eliminating the spherical shape of the abutment, with the aim of reducing the complexity of the model. The finite element partitioning was a compromise between the accuracy of the discretization and the computational possibilities. The loading was the pressure on the surface of these cylinders, so that the total equivalent force was 150 N [23,27,29]. To simulate some clinical aspects, the application of pressure was carried out as follows: on the incisal surfaces of the frontal teeth, a clinical situation specific to the action of cutting food; bilaterally, on the occlusal surfaces of the lateral teeth, a situation that corresponded to the maximum intercuspation position and deglutition; unilaterally, on the occlusal surfaces of the lateral teeth, a situation that corresponded to unilateral chewing action.

**Figure 2:**
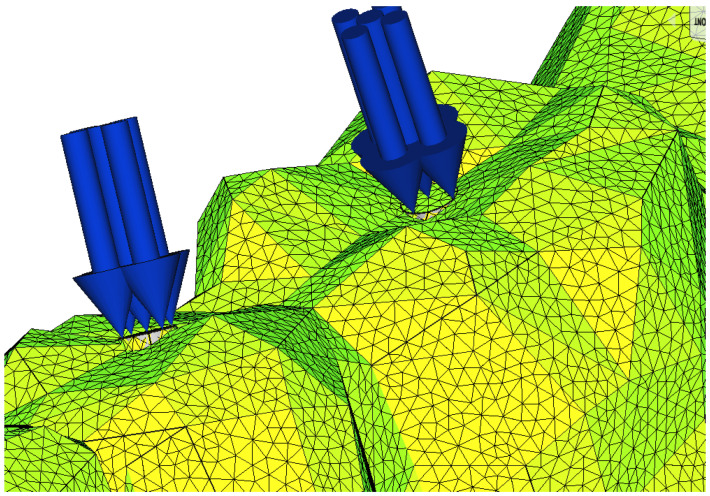
Occlusal loading on the axis of the teeth.

The way in which the loading was carried out was like a pressure on the surface of a small fictitious cylinder. The aim was to avoid the concentrated force effect, and the loading was carried out on the axis of the teeth (Figure 2).

Starting from the initial situation of a mandibular overdenture on four MDIs, with different topographical distributions, several clinical variants that involved the loss of an implant were simulated. The clinical situations were analyzed, depending on the point of insertion of the implants, which were a mandibular overdenture on four interforaminal MDIs (situation 1) and a mandibular overdenture on four MDIs, two of which were inserted in the canine area and two in the molar area (situation 2).

**Figure 3:**
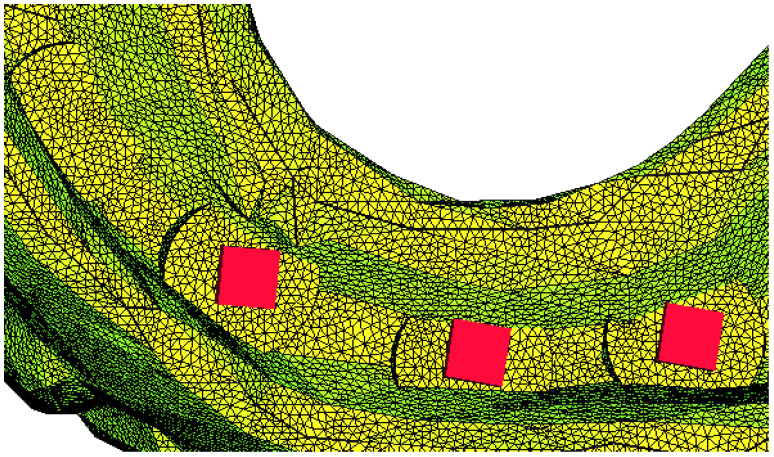
Locking on the surface of three out of four implants.

**Figure 4:**
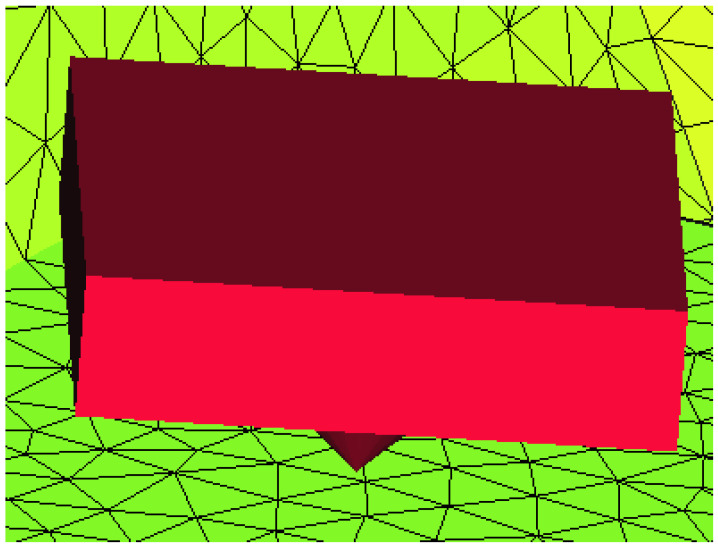
Detail on the locking on the surface of an implant.

In the first situation, the loss of a distal implant was simulated and, for the second situation, the loss of an implant in the molar area was simulated, as well as the loss of an implant in the canine area. After the simulation, the von Mises stress was recorded, with its value being expressed in MPa, as a possible indicator of the risk of fracturing of the overdenture. The displacement, expressed in millimeters, was an indicator of the risk of balance deficiencies emerging in the denture, with a possible negative impact on the support structures and functionality. As a way of applying boundary conditions in displacements, a locking on the surface of the implant was considered (Figure 3 and Figure 4). For a part of the simulations, a hemispherical surface was considered, and later a flat surface was considered, the results being similar, and the model much simpler.

For the analysis, linear elastic and tetrahedral elements were delineated without intermediate nodes, using the freely available, open-source programs GMSH (Geuzaine Christophe and Remacle Jean-Francois, 2020, www.gmsh.info, accessed on 18 March 2022) and CalculiX (Guido Dhondt and Klaus Wittig, www.calculix.de, accessed on 18 March 2022), respectively, for the FEM solutions. The maximum size of an element was about 0.4 mm, and the resulting model contained approximately 200,000 nodes and 1,000,000 volume elements. As a result of rolling, displacements were obtained, and stresses were considered as equivalent von Mises stresses. Different stress levels were highlighted, using a linear and logarithmic scale for the equivalent stresses.

## 3. Results

In this study, the characteristics of the bone or implant components were not considered, and the behavior of the overdenture was analyzed in relation to the number of implants. We aimed to highlight the biomechanical complications of the overdenture after the loss of an implant at different topographic locations, looking at stress concentration and the displacement tendency.

### 3.1. Loading of an Overdenture on four MDIs Inserted in the Interforaminal Area

**Figure 5:**
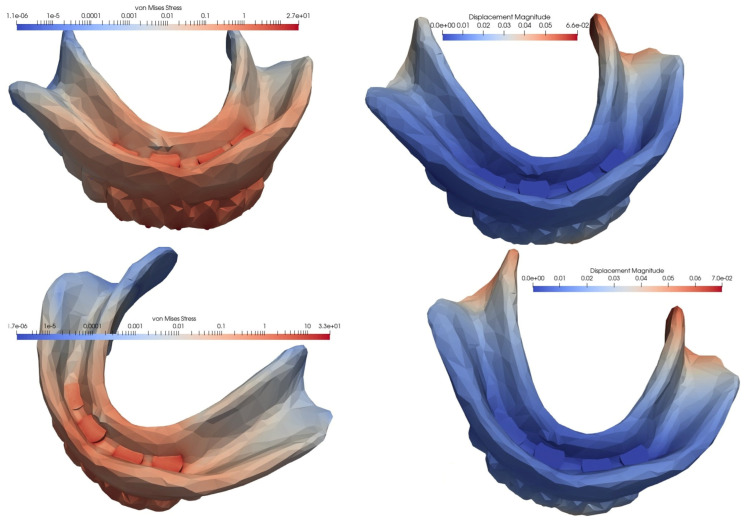
The von Mises stresses and displacements generated by the frontal loading of an overdenture on four interforaminally inserted MDIs (**top**), compared to the loss of a distal implant (**bottom**).

**Figure 6:**
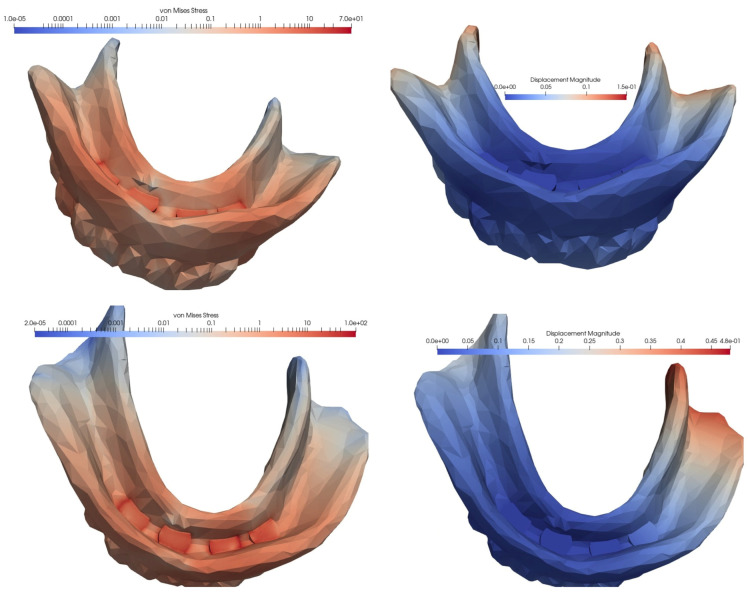
The von Mises stresses and displacements generated by the bilateral loading of an overdenture on four interforaminally inserted MDIs (**top**), compared to the loss of a distal implant (**bottom**).

**Figure 7:**
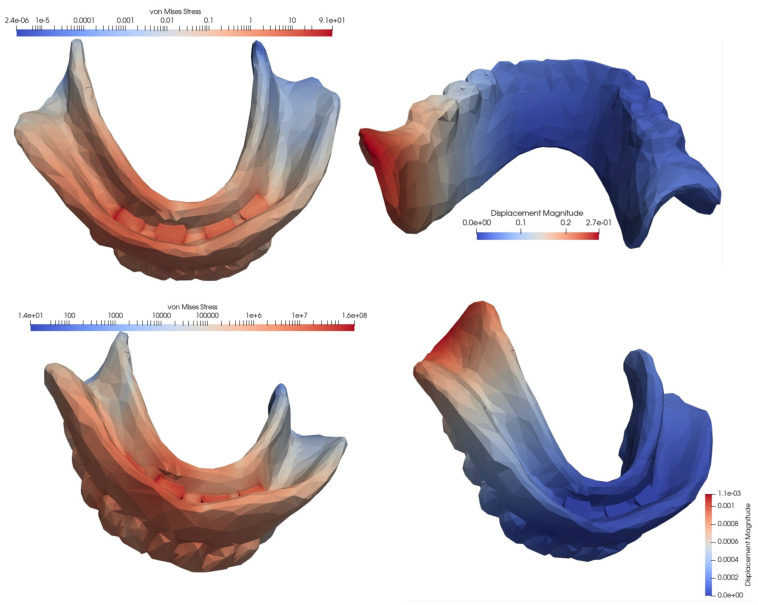
The von Mises stresses and displacements generated by the unilateral loading of an overdenture on four interforaminally inserted MDIs (**top**), compared to the loss of a distal implant (**bottom**).

In the case of an overdenture on four MDIs inserted in the anterior, interforaminal area, the equivalent von Mises stress and the displacement showed the lowest values when the loading was carried out at the level of the frontal teeth, while the highest values appeared in the case of unilateral loading, when the stress was more than three times higher, and the displacement was four times higher. With the loss of a distal implant, increases were observed in von Mises stress and displacement values, but the difference was relatively small when loading was performed in the frontal area, and significantly greater when loading was performed in the lateral area, either unilaterally or bilaterally. Following a comparative analysis of the extreme values, when mastication was performed unilaterally, compared to mastication in the frontal area, the von Mises stress became 4.73 times higher and the displacement was 16.5 times higher (Figure 5, Figure 6 and Figure 7, Table 1).

### 3.2. Loading of an Overdenture on Four MDIs Inserted in the Canine and Molar Areas

In the second simulated situation of a mandibular overdenture on four MDIs, two of which were inserted in the canine area and two in the molar area, the lowest equivalent von Mises stress manifested during frontal loading, and the highest value appeared during unilateral loading, with the tension being over three times higher. In terms of displacement, the lowest value was recorded for bilateral loading, and the highest value for loading in the frontal area: this value was two times as high.

Loss of an implant always resulted in an increase in equivalent von Mises stress, but the amplitude differed depending on the location of the implant and the type of loading. Regardless of the location of the lost implant, tensional values were lower following bilateral loading, and higher after frontal or unilateral loading. Regarding the location of the lost implant, the most important differences in the values of the equivalent stresses were recorded for frontal loading, with the loss of the implant in the canine area being associated with a 4.6 times higher increase, and the loss of the implant in the molar area being associated with a 2.56 times higher increase.

**Figure 8:**
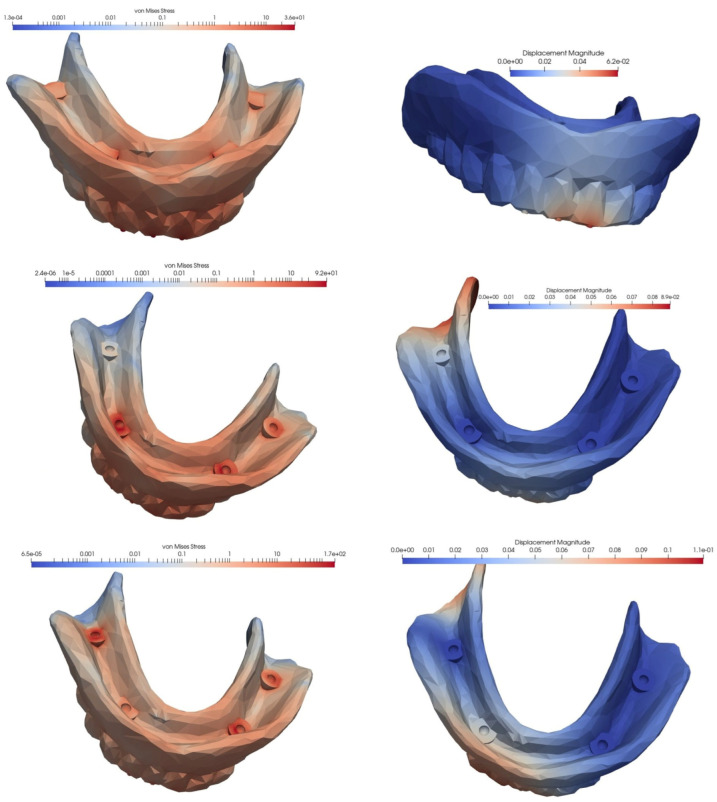
The von Mises stresses and displacements generated by the frontal loading of an overdenture on four MDIs, two of which are inserted in the canine area and two in the molar area (**top**), compared to the situations of loss of an implant in the molar area (**middle**) or the canine area (**bottom**).

**Figure 9:**
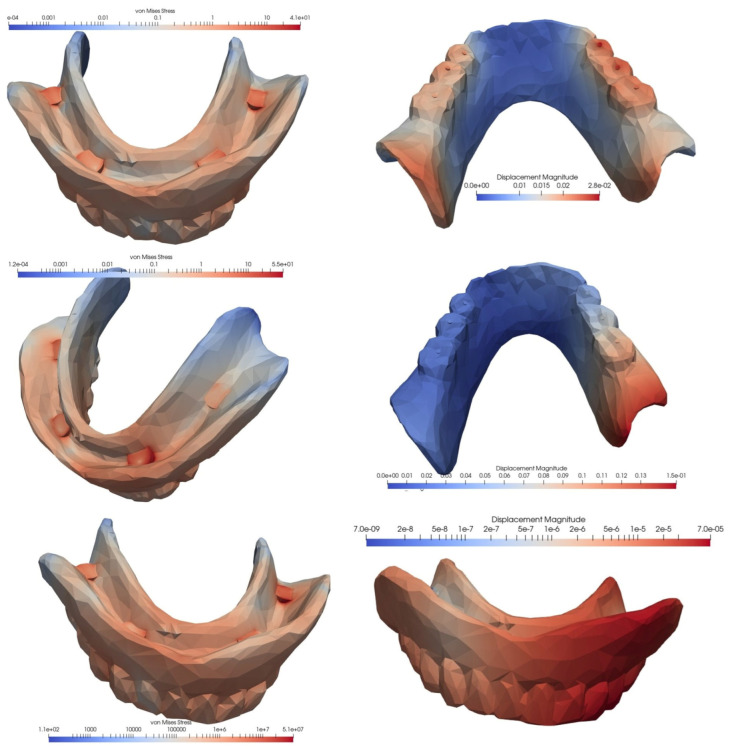
The von Mises stresses and displacements generated by the bilateral loading of an overdenture on four MDIs, two of which are inserted in the canine area and two in the molar area (**top**), compared to the situations of loss of an implant in the molar area (**middle**) or the canine area (**bottom**).

**Figure 10:**
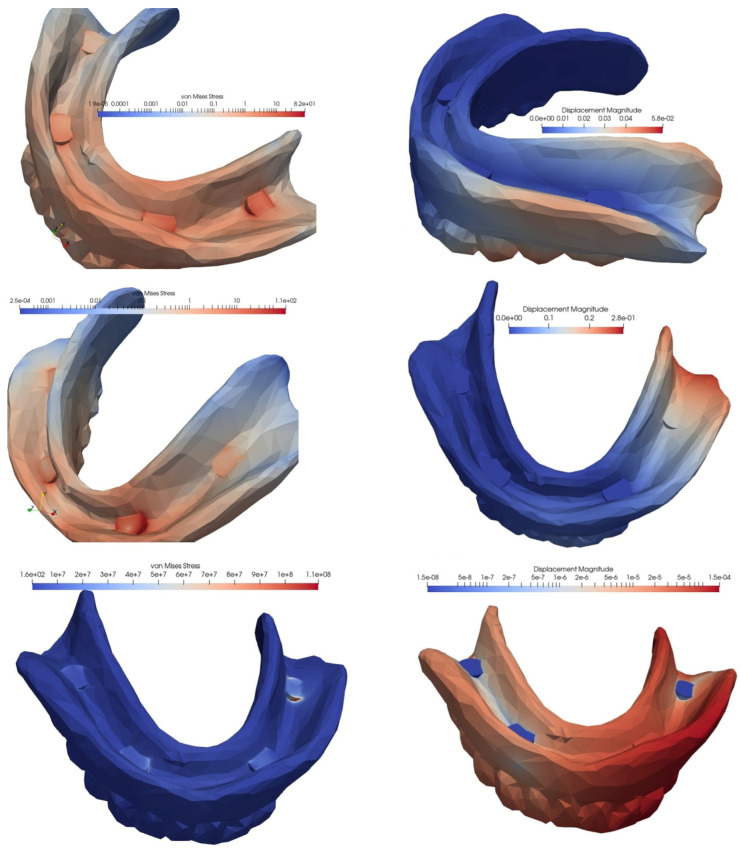
The von Mises stresses and displacements generated by the unilateral loading of an overdenture on four MDIs, two of which are inserted in the canine area and two in the molar area (**top**), compared to the situations of loss of an implant in the molar area (**middle**) or the canine area (**bottom**).

The differences in the amplitude of the displacement were lower in the case of frontal loading and higher in the situation of lateral loading, whether unilateral or bilateral. The loss of an implant from the molar area generated an almost fivefold increase in the displacement value, and was 2.5 times greater when the lost implant was from the canine area (Figure 8, Figure 9 and Figure 10, Table 2).

## 4. Discussion

The von Mises stresses and displacements observed at the level of the mandibular overdenture on four MDIs registered considerable variations in relation to several parameters, such as the place at which the implants were inserted, the topographical location of the lost implant, or the way in which the loading with occlusal forces was carried out: these were the factors according to which a variable change in the amplitude of stresses and displacements was observed in the situation where one of the implants was lost. Thus, the lowest tension values were observed, in the case of the mandibular overdenture, on four MDIs that were inserted interforaminally when loading on the frontal teeth, a situation that simulated the stage of food incision. The lowest displacement was manifested following the bilateral loading of an overdenture on four MDIs, two of which were inserted in the canine area and two in the molar area. At the opposite pole, the highest von Mises stress was observed in the same overdenture, following the loss of an implant inserted in the canine area during frontal loading. The highest displacement was observed for an overdenture on four MDIs that were inserted interforaminally during unilateral loading following the loss of a distal implant.

The loss of one or more implants is a possible mechanical complication of overdentures on implants or MDIs, along with damage to the attachment systems and balance or functional deficiencies of the prosthodontic restorations [15]. Some of the factors that can contribute to the evolution of overdentures complications include the direction and size of the occlusal forces and the degree of bone atrophy, which is correlated with the equilibrium conditions of the dentures. Further contributing factors include the number and topography of the implants, and the anchoring system or type of antagonist [30].

The factors that influence the biodynamic behavior of a mandibular overdenture on MDIs include the prosthetic design [31], the number of MDIs [29], their insertion site and the type of occlusal loading [4]. MDIs have certain advantages compared to conventional implants: studies have demonstrated the occurrence of lower stresses, the smaller diameter and the resilience offered by the attachment system, which allow the comparison of MDIs behavior to a stress breaker [29]. Moreover, the presence of conventional implants or MDIs improves the stability of the denture and, implicitly, functional efficiency in the preparation of the food bowl [32]. The denture design must cover the entire support area of the prosthetic field, and meet all the clinical and technical conditions for the realization of such a prosthodontic restoration; one of the most important parameters to consider is the type of connection with the implant, occlusal scheme and marginal adaptation [31].

Our study demonstrated that the values of von Mises stresses and displacements, following an FEM analysis of different types of mandibular overdentures on four MDIs, varied considerably after the loss of an implant, depending on the location of the lost implant and the type of occlusal loading. The lowest tension values were recorded following loading at the level of the frontal teeth, regardless of the location of the implants, and the highest tensions occurred following unilateral loading. Moreover, unilateral mastication proved to be a higher risk factor than bilateral mastication when implants were also inserted in the lateral area by doubling the values of von Mises stresses. Unilateral loading generally proved to be the most disadvantageous in terms of the appearance of von Mises stresses at the base of the overdentures, near the implants. Additionally, unilateral loading was the worst option regarding displacements, with similar results to those of other researchers [4].

When the implants were inserted interforaminally, the difference between the stresses that appeared between the frontal and lateral areas was much higher than when the implants were also inserted in the lateral area. The displacement’s amplitude increased in the case of unilateral loading of a mandibular overdenture on four MDIs inserted interforaminally, and showed lower values in the case of bilateral loading when the implants were also inserted in the lateral area. In conclusion, the insertion of implants in the lateral area proved to be more beneficial regarding the risk of increased von Mises stresses and displacements.

In terms of the direction, location and intensity of occlusal loading, there were situations in which the von Mises stresses were lower in frontal loading than in bilateral loading, such as in the case of the mandibular overdenture on four interforaminally inserted MDIs, in which all implants were present. In the situation of a mandibular overdenture on four MDIs, inserted in the canine and molar area, we encountered similar results when all four MDIs were present: however, with the loss of one implant, the bilateral loading determined the lowest equivalent stresses. Researchers recommend a bilateral balanced occlusion for overdentures and loading on the most distally inserted implants, in the case of an overdenture on several implants in different locations [4].

While there are studies that do not report a clear influence of the number or type of implants [31], the increase in the number of MDIs in the frontal, interforaminal area, contributed to the reduction in von Mises stresses and displacement values. Therefore, the recommendation of four MDIs instead of two was justified, considering the occlusal stress, and their resistance in relation to the reduced diameter [33]. The researchers demonstrated that an increase in the number of MDIs, and the careful choice of their insertion site at the level of the mandibular ridge, contributed to an improvement in the resistance of the overdentures on four MDIs to lateral or antero-posterior displacement forces, suggesting a greater distance between the anteriorly inserted and the posteriorly inserted MDIs [34].

The insertion of MDIs in the lateral area offered the possibility of special situations in which they would all remain functional, along with the occurrence of less tension and displacement than in the unfavorable situation of losing some of the MDIs. The results differed greatly depending on how the occlusal loading was carried out—either anteriorly, unilaterally or bilaterally—as well as on the location of the remaining MDIs. In this sense, the location of the MDIs was an important factor in the biodynamics of the overdentures, with results differing from the loss of one of the four MDIs. When an MDI was lost in the molar area, in the case of a mandibular overdenture on four MDIs, two of which were inserted in the canine area and two in the molar area, there were fewer displacements following frontal loading than there were in the bilateral or unilateral loading. If the implant that was lost was one of the two in the canine area, then bilateral loading demonstrated the lowest stresses and displacements. To obtain the lowest tensions, biomechanical studies recommend that the canine area is best for the insertion of implants [4], as well as the lateral incisors area [35] or the first premolar area [33].

Possible complications of acrylic dentures also appear in relation to the characteristics of the denture bases’ materials, including their wettability and microbial adhesion [36]. These required a series of interventions by dentists to optimize prosthodontic treatments [37].

Continuous medical care of the patient becomes essential: this takes place through successive prophylaxis sessions, occlusal balancing, the replacement of lost or ineffective attachment systems, and denture linings, when the need arises [15]. Implant systems with spherical abutments, in the case of mandibular overdentures, lead to more frequent complications, due to the larger vertical prosthetic space required and the increased need to adapt the denture base [30]. To reduce the risk of denture fracture, specialists indicate an optimal implant and attachment system, an appropriate number and distribution of implants and retention systems, and a material for the dentures’ base with high impact resistance or the use of a reinforcement system. A correct analysis of the clinical situation was required regarding the available prosthetic space, the type of antagonist, and functional and parafunctional particularities [15].

## 5. Conclusions

An FEM analysis of the clinical situations of implant loss, starting from the mandibular overdenture on four MDIs at different locations, demonstrated the occurrence of changes in the values of the equivalent stresses and displacements of the prosthodontic restoration. These could explain the emergence of complications, such as the instability of the dentures and functional difficulties, as well as an increased risk of fracture of the overdenture. Considering the concentration of equivalent stresses and the risk of displacement of prosthodontic restorations, the bone at the implant insertion site must also be carefully analyzed, in terms of its quantity and quality in relation to dentures’ occlusal stresses and displacement tendencies.

To prevent complications, it is important to comply with all the clinical and technical stages of making the overdentures, such as the choice of an appropriate number of implants and their insertion location, regarding their biomechanical aspects. The choice of a correct occlusal scheme is also important, to ensure optimal functional loadings. Periodic control and occlusal adjustments can reduce unwanted effects and prevent some complications, such as implant loss, displacements or fracture of overdentures.

## Figures and Tables

**Table 1:** Biomechanical changes in the conditions of loss of a distal implant for the mandibular overdenture on four interforaminally inserted MDIs.

	Four MDIs		Three MDIs, after the Loss of a Distal Implant	
Occlusal Loading	von Mises Stress (MPa)	Displacement (mm)	von Mises Stress (MPa)	Displacement (mm)
On the frontal teeth (using the frontal area in mastication)	26.96	0.065	32.91	0.069
On three lateral teeth on both sides (maximum intercuspation and bilateral mastication)	70.41	0.15	103.56	0.48
On three lateral teeth on one side (unilateral mastication)	91.5	0.27	155.72	1.14

**Table 2:** Biomechanical changes under the conditions of the loss of an implant in the canine or molar area for the mandibular overdenture on four MDIs, two of which were inserted in the canine area and two in the molar area.

	Four MDIs		Three MDIs, after the Loss of an Implant in the Molar Area		Three MDIs, after the Loss of an Implant in the Canine Area	
Occlusal Loading	von Mises Stress (MPa)	Displacement (mm)	von Mises stress (MPa)	Displacement (mm)	von Mises Stress (MPa)	Displacement (mm)
On the frontal teeth (using the frontal area in mastication)	35.8	0.061	91.67	0.088	166.1	0.11
On three lateral teeth on both sides (maximum intercuspation and bilateral mastication)	40.87	0.028	55.13	0.15	50.92	0.07
On three lateral teeth on one side (unilateral mastication)	82	0.057	110.66	0.28	113.08	0.15

## Data Availability

The data presented in this study are available on request from the corresponding authors.

## Abbreviations

FEM—Finite element method; MDIs—Mini dental implants.
